# The incentive for innovation and the scientific journals: Could there be conflicts of interest?

**DOI:** 10.5935/0004-2749.2024-1014

**Published:** 2024-10-31

**Authors:** Newton Kara-Junior

**Affiliations:** 1 Ophthalmology Department, Universidade de São Paulo, São Paulo, SP, Brazil

Scientific policies of countries interested in technological development incentivize innovation. In Brazil, *Coordenação de Aperfeiçoamento de Pessoal de Nível Superior* (CAPES) is the government organization that regulates and evaluates *stricto sensu* postgraduate programs and defines research priorities. A key strategy of CAPES is awarding patent registrations as a means to motivate researchers to create novel solutions to pressing problems^(^1^)^.

Although CAPES initiative aligns with Brazils’ developmental goals, its execution has much scope for improvement. The current approach to innovation focuses largely on patent registrations, which provide legal security but do not guarantee an invention’s practical value or societal impact. True innovation encompasses the entire developmental cycle, culminating in a functional product that meets real-world needs and is adopted by people. Thus, CAPES does not necessarily reward the effective result of innovation but rather rewards patent filings without considering the practical outcome. By only acknowledging the beginning of the process, CAPES may inadvertently encourage researchers to prioritize patent filings over the rigorous development and testing required to create viable, socially relevant solutions.

Additionally, CAPES’ policy of incentivizing publications in journals with a high scientific impact factor, which are usually of American and English origin, may not necessarily contribute to Brazil’s technological progression. Often, these articles evaluate the effectiveness of foreign technologies that have recently arrived in the market. This approach can lead to Brazilian researchers using their limited resources to help validate international innovations. The dynamics of international scientific publishing can place Brazilian researchers at a disadvantage. Foreign journals often prioritize the publication of studies that evaluate products developed by their pharmaceutical companies.

However, a significant challenge arises when high--impact journals demand detailed technical disclosures for innovative products as a precondition for publication, enabling its replication. This requirement forces Brazilian researchers to surrender valuable intellectual property, which tantamounts to a free transfer of technology.

In contrast, institutions like the Massachusetts Institute of Technology can afford to disclose the industrial secret of their innovation, as their rapid innovation cycles and abundant resources enable them to stay ahead of competitors. By the time other groups organize to copy their work, researchers are likely already developing the next generation of technology. Brazilian innovators, however, rely heavily on the exclusivity of the original idea to remain competitive due to limited resources.

Publications in high-impact journals facilitate the global dissemination of discoveries, a desirable goal. It is also crucial for scoring points in the CAPES evaluation, which is essential for the postgraduate program. Given this context, national scientific journals need to be indexed in the main electronic databases to also facilitate the dissemination of discoveries across the world. Therefore, to foster national technological innovation, we believe that CAPES needs to value and reward articles describing innovative products, published in national journals.
